# Compliance with standard precautions and associated factors among undergraduate nursing students at governmental universities of Amhara region, Northwest Ethiopia

**DOI:** 10.1186/s12912-022-01165-w

**Published:** 2022-12-30

**Authors:** Desalegn Getachew Ayele, Zewdu Baye Tezera, Negesu Gizaw Demissie, Ashenafi Worku Woretaw

**Affiliations:** 1 Department of Surgical Nursing, School of Nursing, College of Medicine and Health Science, University of Gondar, Gondar, Ethiopia; 2 Department of Comprehensive Nursing, School of Nursing, College of Medicine and Health Science, University of Gondar, Gondar, Ethiopia; 3 Department Medical Nursing, School of Nursing, College of Medicine and Health Science, University of Gondar, Gondar, Ethiopia

**Keywords:** Compliance, Nursing students, Standard precautions

## Abstract

**Background:**

Standard precautions are minimum infection control practices used to prevent the transmission of diseases and applied to all patient care. Nursing students are at high risk of exposure to occupational biologic hazards because they are obligated to provide care to patients admitted with unknown infection statuses. Compliance with standard precautions is an effective and efficient means of infection prevention. However, their compliance with standard precautions among nursing students is not known in Ethiopia. Therefore, this study aimed to assess compliance with standard precautions and associated factors among undergraduate BSc nursing students at governmental universities located in the Amhara Region, northwest Ethiopia.

**Methods:**

An institutional-based cross-sectional study was conducted among undergraduate BSc nursing students at the governmental universities located in Amhara Region, northwest Ethiopia, from April 15 to May 15, 2021. A simple random sampling technique was used to select 423 samples. Descriptive statistics were presented in text, tables, and charts. Multicollinearity and model fitness were checked. All variables were entered into multivariable logistic regression and a *P*-value of < 0.05 was considered to identify statistically significant factors.

**Result:**

Around 221 (53.4%) of the study participants were males. Good compliance of nursing students towards standard precautions was 56.3% (95% CI = 51.4–60.9), which is significantly associated with good knowledge (AOR = 2.52, 95% CI = 1.61–3.94), a perceived safe workplace climate (AOR = 2.15, 95% CI = 1.24–3.71), and training or seminars related to standard precautions in the last six months (AOR = 1.52, 95% CI = 1.01–2.29).

**Conclusion:**

The overall compliance of nursing students with standard precautions was low, with nearly half of the nursing students failing to comply with standard precautions. The major factors associated with good compliance were good knowledge, a perceived safe workplace, and having seminars or training in the last six months. Training, enhancing knowledge, and creating a safe hospital environment are recommended to improve nursing students’ compliance with standard precautions.

## Background

Standard Precautions represent the minimum infection prevention measures that apply to all patient care, regardless of suspected or confirmed infection status of the patient, in any setting where healthcare is delivered [1–3]. It is designed to protect healthcare professionals and patients from exposure to potentially infected blood and body fluids except for sweat [4].

Nowadays, the most common adverse effects in health care worldwide are Health Care-Associated Infections (HCAIs), which endanger the health of both patients and health care staff [5]. Each year,100 million patients and 3 million HCWs are affected with HCAIs [6–8].

Compliance is defined as the extent to which certain health care practices are implemented following known recommendations [9]. Being compliant with standard precautions (SPs) among health care providers reduces the risk of HCAIs by one-third [10]. However, globally, a review revealed that compliance of HCWs with SPs is suboptimal, with a compliance rate of less than 50% [11]. Even if limited studies are found in the world other than Ethiopia among nursing students' compliance with SP, some studies in Croatia, South Korea, and Saudi Arabia revealed that their compliance level is not good [12–15].

Nursing students (NSs) are at high risk of workplace exposure to biological hazards since they are expected to provide treatment for patients of uncertain infection status and due to their underdeveloped abilities and lack of knowledge in the clinical setting [12, 16–19].In particular, they are at high risk of acquiring blood-borne infections, such as HIV infection [20], viral hepatitis [21], and other infectious diseases, like tuberculosis [22]. One study in Ethiopia revealed that more than half and one-third of nursing and midwifery students had needle stick injuries and exposure to blood and other body fluids, respectively [23].

Furthermore, nursing students had prolonged contact with patients and caring than other health science students. Nursing students may have exposed or transmit diseases when they are taking samples, performing wound care, injection, feeding, bathing etc. As a result of their increased activities and contact with patients, we believed that nursing students may have high risk of exposure to blood and other body fluids borne diseases. Due to this reasons we are interested on this groups.

Age, gender, marital status, knowledge, attitude, practicum department, previous sharp and needle stick injuries, blood and other body fluid exposure, work place safety climate, having family working in health teams, and year of study and training on SPs all influence nursing students' compliance with SPs [12–15, 24, 25].

Nursing interventions often require touching the patients, which can facilitate cross-contamination if they fail to comply with proper infection prevention guidelines [26]. Having good compliance with standard precautions protects nursing students from occupational exposure to blood and other body fluid, and lowers the risk of infection transmission to them and patients [27, 28]. However, compliance and associated factors towards standard precautions among undergraduate nursing students in Ethiopia are not known. As a result, this study aims to assess compliance and associated factors towards standard precautions among nursing students in governmental universities of the Amhara region, northwest Ethiopia.

## Methods and materials

### Study design, area and period

An institutional-based cross-sectional study design was conducted from April 15 to May 15, 2021, at governmental universities located in Amhara region, northwest Ethiopia,2021. Amhara region is one of the ten regions in Ethiopia and is located in the Northwest part of the country Ethiopia. Its Capital city is Bahir Dar, which is located 565 km from Addis Ababa, the capital city of Ethiopia. There are 10 governmental universities in the region. Among these universities, Bahir Dar (BDU), Debretabor (DTU), Debre Markos (DMU), and the University of Gondar (UOG), which have a college of health science and teach nursing students, and were included in the study. There were a total of 738 third- and fourth-year nursing students at selected universities. However, first-year and second-year nursing students were not yet joining the universities and the nursing schools because of the COVID-19 interruption of the learning-teaching process in Ethiopia.

### Source population and study population

#### Source population

All third-year and fourth-year undergraduate nursing students who were learning at the four Governmental Universities located in Amhara Region, northwest Ethiopia.

#### Study population

All third-year and fourth-year undergraduate nursing students who were learning at the four Governmental Universities located in Amhara Region, northwest Ethiopia and available during the data collection period.

### Inclusion and exclusion criteria

All third-year and fourth-year undergraduate nursing students who were available during data collection time.

### Sample size determination and sampling technique

#### Sample size determination

The sample size was determined by using single population proportion formula using 95% confidence level (Z = 1.96), degree of precision (marginal error) = 5%, and proportion (*p* = 50% since there was no previous study in Ethiopia).

The calculated final sample size was 423 including 10% none response.

#### Sampling technique

**Fig. 1:**
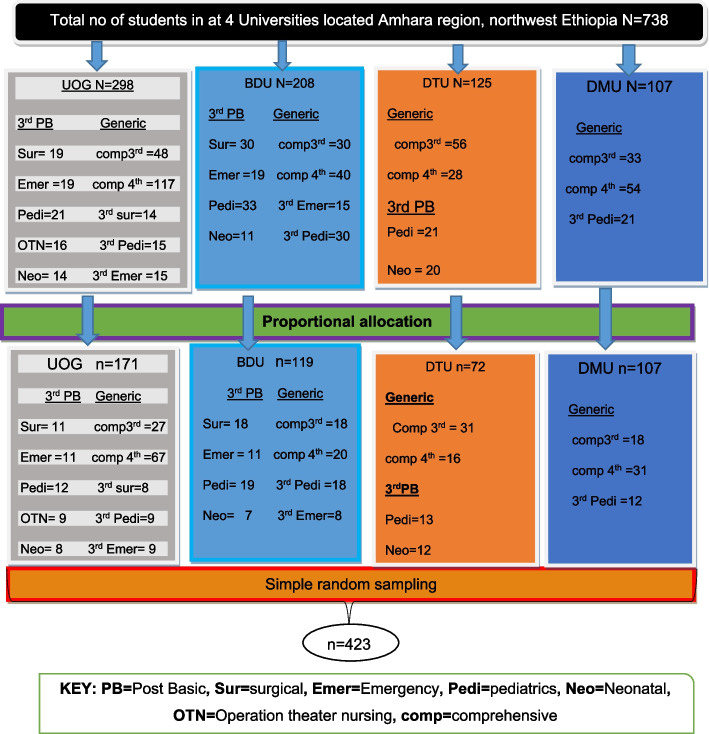
Schematic presentation of the sampling procedure on compliance with standard precautions and associated factors among nursing students at Amhara region, northwest Ethiopia, 2021 (*n* = 414)

The list of nursing students was taken from the four universities’ nursing departments. For each university, proportionate allocation was used, with UoG = 171, DTU = 72, BDU = 119, and DMU = 61. For each academic year and program at each university, a proportional allocation was used. Then, from each academic year and program, each class was given a proportionate allocation. Finally, participants from each class were chosen using a simple random sampling technique (Fig. 1).

### Study variables

#### Dependent variables

Compliance with standard precaution.

#### Independent variable

**Socio-demographic** characteristics such as

- ➢ Age, marital status, gender, year of study, previously working as a nurse, family working in the healthcare team, learning department (specialty), and program.

##### Personal and Institutional factors

Knowledge, Attitude, previous needle stick injury, and blood and other body exposure, Workplace Safety climate, practicum department, training or seminar.

### Operational definitions

**Compliant (Good compliance);** if the participant scores greater than or equal to the median (11) score of compliance questions [28].

**Noncompliant (Poor compliance):** if the participant scores less than the median (11) score of compliance questions [28].

**Good knowledge**: if the participant scores greater than or equal to the median (7) score of knowledge questions [29].

**Poor knowledge:** if the participant scores less than the median (7) score of knowledge questions [29].

**Positive attitude:** if the participant scores greater than or equal to the median (22) score of attitude questions [29].

**Negative attitude:** if the participant scores less than the median (22) score of attitude questions [29].

**Safe workplace climate:** if the participant scores greater than or equal to the median (5) score of safety questions.

**Unsafe workplace climate:** if the participant scores less than the median (5) score of safety questions.

### Data collection tool and procedure

Data were collected using a self-administered structured questionnaire to obtain information from participants. The questionnaire is divided into three parts. Part I asked for Socio-demographic variables and had 13 questions. Part II has the Compliance with Standard Precautions Scale (CSPs). The CSPS is a 20-item scale that assesses self-reported compliance with SPs. The scale’s items evaluate compliance with the use of PPE (6 questions), disposal of sharps (3 Questions) disposal of wastes (1 question), decontamination of spills and used articles (3 questions), and prevention of cross-infection (7 questions). The response set is a 4-point Likert scale that consists of responses such as (‘‘never’’, = 1 ‘‘seldom’ = 2’sometimes’’ = 3, and ‘‘always’’ = 4 during data collection.it was also recoded into 1 and 0. A score of 1 is interpreted as an ‘‘always’’ response, while 0 is applied for the other responses. A total range score of 0—20 is expected, with higher scores signifying better compliance with SPs. Items 202, 204, 206, and 215 are negatively stated; thus, scores were reversed before computations.as (‘‘never’’, = 4 ‘‘seldom’ = 3’sometimes’’ = 2, and ''always'' = 0. Then it was also recoded to 1 and 0. A score of 1 is interpreted as an ‘‘never’’ response, while 0 is applied for the other responses. The internal consistency of the original tool was checked and Cronbach’s α value was 0.73. The tool is adapted from Hong Kong [28]. Part III had questions of factors affecting compliance of nursing students, and it has three sections. Section I, Knowledge questions, had 10 multiple choice questions, Section II is attitude questions, had 7 questions with 5 points Likert scale (1 = strongly disagree,2 = disagree, 3 = Undecided4 = Agree and 5 = strongly agree. Section III is safety climate questions, had 7 yes or no responses. Knowledge, attitude, and safety climate questions were adapted from tools used for HCWs in Ethiopia and Korea [29, 30]. study participants were approached in each ward unit.

Eligible nursing students in specified classrooms were selected based on inclusion criteria, after getting informed consent the data collector was administered the questionnaire. Participants were provided with appropriate information about the study, then informed consent was being obtained to assure their willingness to participate in the study. Four trained BSc nurses collected the data and four trained MSc nurses closely followed the data collection process.

### Data quality assurance

To ensure the quality of data one-day training was given to data collectors and supervisors regarding the structured tool (on the objective of the study and how to collect the data). A week before starting the actual period, the questionnaire was pretested in Woldia University on 10% of the total sample size of nursing students, and the necessary correction was done. Internal consistency was checked by computing Cronbach’s α for the dependent variable. It was 0.709 from the pretest data. Regular daily supervision was done to check, the consistency and completeness of the filled-out checklist format, by the principal investigator and supervisors.

### Data processing and analysis

Data were coded and entered into Epi-info version 7 and then it was exported to SPSS version 25 for analysis. Data entry was made by the principal investigator with close supervision of coauthors. Descriptive statistics including, frequencies, proportions, mean, median and SD was computed and displayed by using tables, graphs, and texts. The model fitness was checked by Hosmer and Lemeshow’s goodness of fit test and its *p*-value was 0.716. Multicollinearity was checked.

All variables were entered in to multivariable logistic regression analysis examine the association between the dependent variable and independent variables. Those with *p* < 0.05 at multivariable logistic regression analysis were considered statistically significant.

## Results

Among 414 study participants, 56.3% with 95%CI (51.4,60.9) of them were compliant with standard precautions and the rest were non-compliant.

### Sociodemographic characteristics of the study participants

The data were collected from a total of 414 randomly selected participants with a response rate of 97.8%. Of the total respondents, 221 (53.4%) were males. The median age of the participants was 24 with an IQR = 6 years. Of the 414 nursing students, 287(69%) were single. More than two-thirds of nursing students, 283 (68.4%) were third year and 286 (69%) learned generic program. Around 211(50%) of the nursing students were from comprehensive nursing department followed by pediatrics nursing department. Majority of the nursing students’ parents 359 (86.7%) did not work in the health care team (Table 1).

**Table 1:** Frequency distribution for socio-demographic characteristics of nursing students at governmental universities in Amhara region, Northwest Ethiopia, 2021 (*n* = 414)

Variables	Category	Frequency	Percent
Sex	**Male**	221	53.4
	**Female**	193	46.6
Age	median $\pm$ IQR	24 $\pm$ 6	
Marital status	**Single**	287	69
	**Married**	127	31
Years of study	**Third**	283	68.4
	**Fourth**	131	31.6
Learning program	**Generic**	286	69
	**Post Basic**	128	31
Department(specialty)	**Surgical**	36	8.7
	**Pediatrics**	89	21.5
	**operation theater**	11	2.7
	**Emergency**	40	9.7
	**Neonatal**	27	6.5
	**Comprehensive**	211	51
Does your father or mother work in the healthcare team	Yes	55	13.3
	No	359	86.7

### Personal and Institutional characteristics

In this study, one–third (32.4%) of nursing students had good knowledge towards standard precautions, and 54.3% of them had a positive attitude. A total of 192(46.4%) nursing students were reported that they had training or seminar on standard precautions in the last six months, and 383 (82.9%) of the participants were attached to the pediatric ward. Majority 334 (80.7%) of study participants perceived their work place climate as unsafe. More than half of the nursing students 235 (56.8%) did not worked in a health care setting as a nurse before registering for this degree. Nearly two third of study participants had history of exposure to blood and other body splashes and nearly one fourth (25.1%) of the nursing students had ever exposed to needle stick injury during their clinical practice. Around 85% of the nursing students had clinical practicum(attachment) in pediatrics ward (Table 2).

**Table 2:** Frequency distribution for personal and institutional factors of nursing students compliance with standard precautions at governmental universities located in Amhara region, Northwest Ethiopia, 2021 (*n* = 414)

Variables	Category	Frequency	Percentage(%)
Have you worked in a health care setting as a nurse before registering for this degree	Yes	179	**43.2**
	No	235	56.8
Have you ever exposed blood and other body splashes	Yes	149	**36**
	No	275	64
Have you ever exposed to needle stick injury in your clinical practice	Yes	104	**25.1**
	No	310	74.9
Knowledge	Good	134	**32.4**
	Poor	280	67.6
Attitude	Positive	225	**54.3**
	Negative	189	45.7
Have you training or seminar on standard precautions in the last six months	Yes	192	46.4
	No	222	53.6
Workplace safety climate	Safe	80	**19.3**
	Unsafe	334	80.7
Practicum ward	Surgical	266	64.3
	Pediatrics	343	**82.9**
	Medical	222	53.6
	Emergency	255	61.6

### Factors associated with compliance with standard precautions

Having good knowledge (AOR = 2.52, 95% CI = 1.61–3.94), Having training or seminar related to standard precautions in the last six months (AOR = 1.52, 95% CI = 1.01–2.29), and Participants perceived workplace climate as safe (AOR = 2.57, 95% CI = 1.24–3.71) were significantly associated with their compliance (Table 3).

**Table 3:** Shows the Bivariate and Multivariate analysis of factors associated with nursing student's compliance with standard precautions at governmental universities located in Amhara region, Northwest Ethiopia, 2021 (*n* = 414)

Variables	Compliance level		COR (95%CI)	AOR (95%CI)	*P*-value
	Compliant N (%)	Non-compliant N (%)			
**Gender of the respondent**					
Male	106 (25.6)	87 (21)	0.90 (0.61–1.31)	1.18(0.77–1.81)	0.44
Female	127 (30.7)	94 (22.7)	1	1	
**Age of respondent**	Continuous		1.04 (0.99–1.09)	1.04 (0.97–1.11)	0.29
**Marital status**					
Married	77(18.6)	50(12.1)	1.31 (0.85–1.99)	1.048 (0.61–1.81)	0.87
Single	155(37.4)	132(31.9)	1	1	
**Study year**					
4^th^ year	66(15.9)	65(15.7)	0.71 (0.46–1.07)	0.728(.47–1.12)	0.15
3^rd^ year	167(40.3)	116(28.1)	1	1	
**Program**					
Post basic	77(18.6)	51(12,3)	1.26 (0.82–1.92)	1.019 (0.62–1.68)	0.94
Generic	156(37.7)	130(32.4)	1	1	
**Nursing Specialty**					
Surgical	22(5.3)	14(3.4)	1.22 (0.59–2.54)	0.598 (0.23–1.57)	0.0.3
Pediatrics	50(12.1)	39(9.4)	0.99 (0.60–1.63)	0.41 (0.20–0.84)	0.16
ORT	6(1.4)	5(1.2)	0.93 (0.28–3.13)	0.32 (0.08–1.37)	0.13
Emergency	22(5.3)	18(4.3)	0.95 (0.48–1.87)	0.37 (0.15–0.92)	0.32
Neonatal	14(3.4)	13(3.1)	0.83 (0.37–1.86)	0.31 (0.1–0.95)	0.4
Comprehensive	119(28.7)	92(22.2)	1	1	
**Have you worked in a healthcare setting as a nurse before registering for this degree?**					
Yes	107(30.7)	72(17.4)	1.286 (0.87–1.91)	1.157 (0.58–2.3)	0.67
No	126 (30.4)	109(21.5)	1	1	
**Have you ever exposed to needle stick injury in your clinical practice?**					
Yes	60(14.5)	44(10.6)	1.08 (0.69–1.69)	1.23 (0.68–2.25)	.49
No	173(41.8)	137(30.1)	1	1	
**Have you training or seminar in the last six months**					
Yes	121(29.2)	71(17.1)	1.67 (1.13–2.48)	1.518 (1.01–2.29)	0.046*
No	112(27.1)	110(25.6)	1	1	
**Have you ever exposed to blood or other body fluids in your clinical practice**					
Yes	84(20.3)	65(15.7)	1.01 (0.67–1.51)	0.72 (0.46–1.15)	0.17
No	149(36)	116(28)	1	1	
**Does your father or mother worked in healthcare team**					
Yes	31(7.5)	24(5.8)	1.01 (0.57–1.78)	1.08 (0.57–2.05)	0.81
No	202(48.8)	157(37.9)			
**Knowledge of respondents**					
Good	95(22.9)	39(9.4)	2.51 (1.61–3.89)	2.519 (1.61–3.94)	000*
Poor	138(33.4)	142(34.3)	1	1	
**Attitude of respondents**					
Positive	123(29.7)	102(24.6)	0.87 (0.59–1.28)	0.92 (0.6–1.43)	.72
Negative	110(26.6)	79(19.1)	1	1	
**Perceived workplace safety climate**					
Safe	57(13.8)	23(5.6)	2.23 (1.31–3.78)	2.15 (1.24–3.71)	006*
Unsafe	176(42.5)	158(38.1)	1	1	
**Practicum ward**					
Surgical					
Yes	147(35.5)	119(28.7)	1.12 (0.75–1.69)	2.684 (0.72–10.04)	0.14
No	86(20.8)	62(15)	1	1	
Emergency					
Yes	141(34.1)	114(27.5)	1.11 (0.74–1.66)	1.76 (0.68–4.57)	0.24
No	92(22.2)	67(16.2)	1	1	
Medical					
Yes	121(29.2)	101(24.4)	1.15 (0.79–1.73)	0.74 (0.43–1.3)	0.29
No	112(21.1)	80(19.3)	1	1	
Pediatrics					
Yes	186(44.9)	157(37.9)	1.65 (0.97–2.82)	1.42 (0.78–2.58)	0.25
No	47(11.4)	24(5.8)	1	1	
Gyn/obs					
Yes	169(40.8)	142(34.3)	1.38 (0.87–2.18)	0.95 (0.45–2.01)	0.9
No	64(15.5)	39(9.4)	1	1	

1= represents reference group

*Variable significant at *p*-value less than 0.05

## Discussion

This cross-sectional study assessed the self-reported compliance with SPs and the factors associated with it among nursing students. Findings of the current study showed that 56.3% with 95%CI (51.4,60.9) of the study participants were compliant with standard precautions. Good Knowledge, perceived workplace safety, and having training or seminar in the last six months were identified factors significantly associated with being compliant with standard precautions.

The compliance of nursing students towards SPs in this study is in line with studies conducted in Hong Kong 53.5% [28],in Croatia 58.4% [15] and in Saudi Arabia 60.1%, and 56.8% [13, 25] and in Nigeria 57.3 [31]. Even though there is a difference in socioeconomic status and level of health sector development, the possible reason for the similarity might be the use of a similar tool, characteristics of study participants (inclusion of nursing students senior nursing students), and study design.

The finding was lower than studies conducted in South Korea 79.74% [32], 85% in Malaysia [33], Saudi Arabia 61%, and 84.8% [24, 34]. The possible reason might be the difference in a hospital setting, sampling techniques, study population characteristics, the availability and accessibility of safety materials (clinical environment), curriculum, and socioeconomic differences. For example, in South Korea sampling techniques were convenient and included only final year nursing students. Additionally, there might be differences in teachers' close monitoring and following up of students during their clinical practices.

However, The result of this study was higher than a study conducted in South Korea 50.5% [14] and in Egypt, 15% of them had good compliance [35]. This difference might be the study conducted in South Korea is in a single setting where the current study is a multicenter study and in Egypt sampling technique and sample size difference.

In this study the maximum compliance was on putting used sharp articles into sharps boxes. which is consistence with studies conducted in South Korea [19] and Whereas the lowest compliance in this study was disposing sharps box only when it is full, which is consistent with studies conducted in Croatia [20] and Saudi Arabia [30, 31].

Study participants with good knowledge were found to be 2.52 times more likely to comply with standard precautions as compared to nursing students with poor knowledge. This finding is consistent with the study done in Melanesia [36], China [19] and Iran [37], and Nigeria [38]. The possible explanation could be, knowledge is a pre-requisite to appropriate behavioral change and a very important element for behavior change [39]. This is also supported by literature that lack of knowledge is the major reason for non-compliance to standard precautions measures [40]. So having good knowledge helps to implement standard precautions measures properly as recommended. On the other hand, the finding of the current study Contradicts with the studies in the Philippines [41], Malaysia [33], and South Korea [32] showed that no association between knowledge and compliance.

In the current study participants who had training or seminar related to standard precautions were found to be 1.52 times more likely to be compliant with standard precautions than those who had not taken any training or seminar in the last six months. The current result is consistent with previous literatures in Hong Kong, Saudi Arabia, and Jordan showed that individuals with proper training and education seminar-workshop in infection control are more compliant [19, 24, 25, 42]. This might be justified as the fact that training and seminars can sensitize the knowledge of nursing students make them to comply with standard precaution measures. Which also supported by CDC recommendations that training is required for all health care providers to maintain competency and ensure that infection prevention policies and procedures are understood and followed [1].

Participants who perceived workplace climate as safe were found to be 2.15 times more likely to be compliant with standard precautions than those who perceived the workplace as unsafe. This finding is consistent with the studies conducted in South Korea revealed that the higher the perception of a safe environment for standard precautions results the higher compliance with IPC practices [30, 32]. The possible explanation could be well equipped and a safe environment is mandatory to accomplish tasks according to recommendations. Since Safe workplace climate is the shared perception of management for safety support and feedback regarding infection prevention and control in hospitals, including a supportive work environment as well as adequate infrastructure and resources [43]. So the health facilities' infection prevention climate needs to be improved to increase students’ compliance with standard precautions.

## Conclusion

Generally, nursing students’ compliance with standard precautions was low as compared with CDC and WHO recommendations. Good Knowledge, perceived workplace safety, and having training or seminar in the last six months were significantly associated with being compliant with standard precautions. Great emphasis is required from universities, hospitals as well as policy makers.

## Strength and limitation

The use of a self-administered questionnaire may overestimate or underestimate the result of this study. On the other hand, Since It is the first study in Ethiopia among nursing students and it tried to show their compliance clearly.

## Abbreviations

AOR: Adjusted Odds Ratio
BSc: Bachelor of Science
CI: Confidence Interval
COR: Crude Odds Ratio
CSPs: Compliance with Standard Precautions Scale
Epi- Info: Epidemiological Information
HAIs: Hospital-Acquired Infections
HBV: Hepatitis B Virus
HCAIs: Health Care-Associated Infections
HCWs: Health Care Workers
IPC: Infection Prevention and Control
NSs: Nursing Students
PPE: Personal Protective Equipment
SPs: Standard precautions
SPSS: Statistical Package software for Social Sciences
SPSS: Statistical Package for Social Science
WHO: World Health Organization
