# Correction to: Compliance with standard precautions and associated factors among undergraduate nursing students at governmental universities of Amhara region, Northwest Ethiopia

**DOI:** 10.1186/s12912-023-01221-z

**Published:** 2023-03-13

**Authors:** Desalegn Getachew Ayele, Zewdu Baye Tezera, Negesu Gizaw Demissie, Ashenafi Worku Woretaw

**Affiliations:** 1 Department of Surgical Nursing, School of Nursing, College of Medicine and Health Science, University of Gondar, Gondar, Ethiopia; 2 Department of Comprehensive Nursing, School of Nursing, College of Medicine and Health Science, University of Gondar, Gondar, Ethiopia; 3 Department Medical Nursing, School of Nursing, College of Medicine and Health Science, University of Gondar, Gondar, Ethiopia

**Correction to: BMC Nursing (2022) 21:375**

10.1186/s12912-022-01165-w

Following publication of the original article [1], the authors reported errors in the second and the third author names.

The original author names were:

Zewdu BayeTezera^2^, Negesu Gizaw Demssie^3^

The correct author names are:

Zewdu Baye Tezera^2^, Negesu Gizaw Demissie^3^

The correct author names have been provided in this Correction. The original article [1] has been corrected.
